# Identification of DKK-1 as a novel mediator of statin effects in human endothelial cells

**DOI:** 10.1038/s41598-018-35119-7

**Published:** 2018-11-12

**Authors:** Marta Pontremoli, Maura Brioschi, Roberta Baetta, Stefania Ghilardi, Cristina Banfi

**Affiliations:** Centro Cardiologico Monzino, IRCCS, Milano, Italy

## Abstract

This study shows that DKK-1, a member of the Dickkopf family and a regulator of the Wnt pathways, represents a novel target of statins which, through the inhibition of HMG-CoA reductase and of non-steroidal isoprenoid intermediates, exert extra-beneficial effect in preventing atherosclerosis beyond their effect on the lipid profile. We found that atorvastatin downregulates DKK-1 protein (−88.3 ± 4.1%) and mRNA expression (−90 ± 4.2%) through the inhibition of Cdc42, Rho and Rac geranylgeranylated proteins. Further, a combined approach based on the integration of label-free quantitative mass spectrometry based-proteomics and gene silencing allowed us to demonstrate that DKK-1 itself mediates, at least in part, statin effects on human endothelial cells. Indeed, DKK-1 is responsible for the regulation of the 21% of the statin-modulated proteins, which include, among others, clusterin/apoJ, plasminogen activator inhibitor type 1 (PAI-1), myristoylated alanine-rich C-kinase substrate (MARCKS), and pentraxin 3 (PTX3). The Gene Ontology enrichment annotation revealed that DKK-1 is also a potential mediator of the extracellular matrix organization, platelet activation and response to wounding processes induced by statin. Finally, we found that plasma level of DKK-1 from cholesterol-fed rabbits treated with atorvastatin (2.5 mg/kg/day for 8 weeks) was lower (−42 ± 23%) than that of control animals. Thus, DKK-1 is not only a target of statin but it directly regulates the expression of molecules involved in a plethora of biological functions, thus expanding its role, which has been so far restricted mainly to cancer.

## Introduction

DKK-1, a member of the Dickkopf family, was originally identified in Xenopus as an inhibitor of β-catenin-dependent Wnt signalling and an inducer of head formation during embryogenesis, a phenotype that coined the Dickkopf (German for ‘big head, stubborn’) nomenclature^1^. Its human homologue was also characterised as a potent Wnt inhibitor^2^. Thereafter, multiple studies demonstrated that DKK-1 impeded β-catenin-dependent Wnt signalling by binding to the LRP6 co-receptor with high affinity and blocking signalling^3,4^. More recently, DKK-1 has also been proposed to activate β-catenin-independent Wnt pathways through a mechanism that is not well known but is probably indirect and involves DKK-1 shifting the Wnt signaling balance from the β-catenin-dependent pathway to β-catenin-independent pathways^5^. Therefore, the effect of DKK-1 on cellular function is rather complex and presumably involves modulation of both canonical and non-canonical Wnt pathways^5^. Based on the ability of DKK-1 to inhibit β-catenin-dependent Wnt signaling, a pathway that is frequently dysregulated in cancer cells, DKK-1 has been widely investigated in oncology and is now considered an attractive therapeutic target for anti-cancer therapy^5^. However, the importance of DKK-1 and the other members of the DKK family in the pathophysiology of the arterial wall is beginning to be understood.

Ueland *et al*.^6^ reported increased levels of DKK-1 in experimental and clinical atherosclerosis, both systemically and within the atherosclerotic lesion, with particularly high levels in advanced and unstable disease. They showed that platelets and endothelial cells (ECs) are important cellular sources of DKK-1, and provided evidence suggesting that platelet- and endothelial-derived DKK-1 contributes to the inflammatory cross-talk between these cells by inhibiting the canonical β-catenin-dependent Wnt pathway and enhancing NF-kB activation^6^. In line with these findings, plasma DKK-1 levels were found to be significantly higher in patients with type 2 diabetes mellitus (T2DM) compared to healthy subjects, in association with increased levels of endothelial dysfunction and platelet activation markers. Moreover, DKK-1 was found to be downregulated by ameliorating glycemic control and/or by inhibiting platelet function by low-dose aspirin treatment, which support its involvement in the inflammatory interaction between platelets and ECs in the setting of T2DM^7^. In addition, elevated concentrations of DKK-1 were observed in patients with atherosclerotic disorders^8–10^ and in subjects with symptomatic aortic stenosis^11,12^. Moreover, analyses from the prospective population-based Bruneck Study displayed a significant positive association between the plasma level of DKK-1 and the common carotid artery intima-media thickness (IMT), a surrogate marker of early atherosclerosis^13^.

Data from animal atherosclerosis studies and *in vitro* investigations support the observations in humans, suggesting that DKK-1 may be an attractive target for cardiovascular therapy. Di *et al*. reported that silencing of DKK-1 in ApoE^−/−^ mice attenuated atherogenesis^14,15^, while overexpression of DKK-1 by means of lentivirus transfection promoted lesion formation and vulnerability and increased EC apoptosis^15^. In addition, they showed that DKK-1 expression in human umbilical vein endothelial cells (HUVECs) is upregulated by two important proatherogenic factors, perturbation of laminar shear stress^14^ and exposure to oxidized low-density lipoprotein^15^, and demonstrated that silencing of DKK-1 prevented the detrimental effects of these factors on endothelial cell function. Cheng *et al*.^16^ reported that transduction of cultured aortic bovine ECs with vectors expressing DKK-1 inhibits the expression of EC differentiation markers and promotes endothelial-mesenchymal transition (EndMT), a process of cellular trans-differentiation that has emerged as an important driver of atherosclerosis progression and vascular calcification^17,18^. In addition, it has been demonstrated that platelet-derived DKK-1 has a unique proinflammatory role since it potentiates neutrophilic infiltration during the innate immune response^19^ and promotes pathological type-2 cell-mediated inflammation^20^. Overall, these findings suggest that antagonizing DKK-1 pathway could be a promising strategy in atheroprotection.

We and others have previously shown that inhibition of the mevalonate pathway by statin treatment decreases the expression of DKK-1 in cultured cells, specifically in the immortalised endothelial hybrid cell line EA.hy. 926 and in cancer cells^21–23^. Herein, we provide evidence that DKK-1 is indeed a target of statin in human primary vascular cells and, by means of a quantitative label-free proteomic approach, we showed that it could mediate several of the pleiotropic effects exerted by this multifaceted class of drugs^24^.

## Results

### Atorvastatin down-regulates DKK-1 biosynthesis in human ECs by inhibiting protein geranylgeranylation

**Figure 1:**
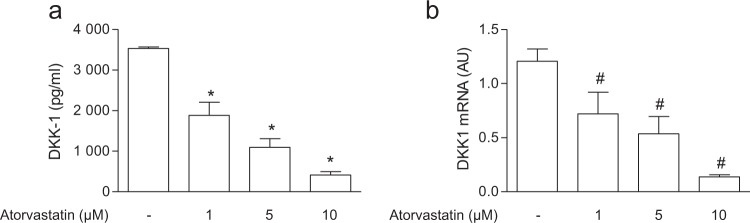
Atorvastatin reduces DKK-1 antigen release (**a**) and mRNA (**b**) in human endothelial cells. Data are expressed as mean ± SEM of 3 individual experiments performed in duplicate. AU, arbitrary units. *p < 0.001 vs control cells; ^#^p < 0.05 vs control cells.

**Figure 2:**
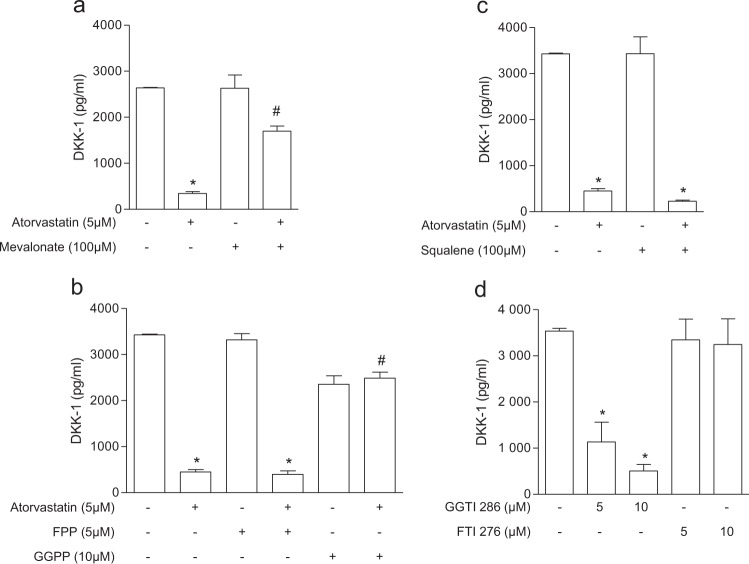
DKK-1 downregulation occurs via geranylgeranylation inhibition. DKK-1 antigen was analysed in HUVECs treated with atorvastatin alone and in combination with mevalonate (**a**), GGPP and FPP (**b**), or squalene (**c**), and in HUVECs treated with the prenyltransferases inhibitors, GGTI-286 and FTI-277 (**d**). Values are the mean ± SEM of 3 individual experiments. *p < 0.01 *vs* control cells; ^#^p < 0.01 *vs* statin-treated cells.

**Figure 3:**
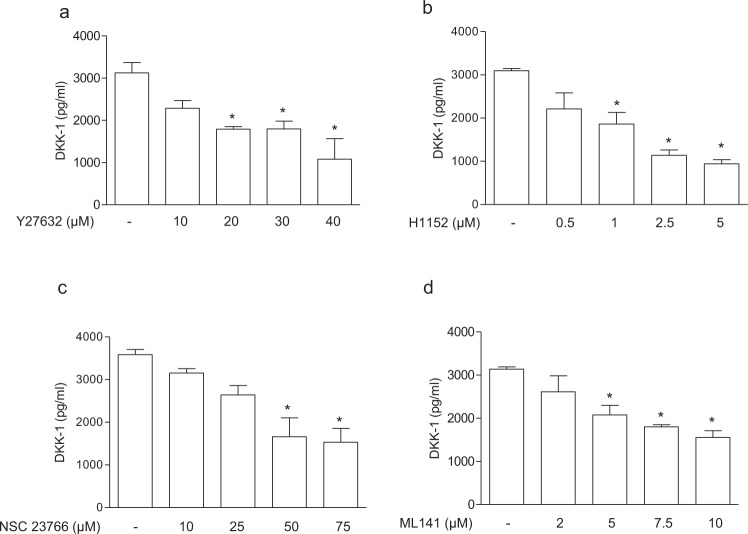
DDK1 release is regulated by geranylgeranylated proteins. DKK-1 antigen was analysed in HUVECs treated with the Rho-associated protein kinase inhibitors, Y-27632 (**a**) and H-1152 (**b**), the Rac1/2 inhibitor, NSC23766 (**c**), and the Cdc42 inhibitor, ML-141 (**d**). Values are the mean ± SEM of 3 individual experiments. *p < 0.05 *vs* control cells.

Based on the previous observation that statins decrease DKK-1 in the secretome of the immortalised endothelial hybrid cell line EA.hy. 926, established by fusing a human umbilical vein endothelial cell with a human carcinoma cell line^21,25^, we addressed the effect of atorvastatin in the primary human endothelial cells HUVECs. We found that atorvastatin reduced DKK-1 biosynthesis in a concentration-dependent manner (Fig. 1). DKK-1 release was reduced also in human aortic smooth muscle cells (AoSMCs) (Supplementary Fig. 1A). To determine whether the effects of atorvastatin on DKK-1 was caused by inhibition of the mevalonate pathway, we evaluated the ability of mevalonate and its isoprenoid derivatives to counteract the inhibitory effect of atorvastatin on DKK-1. Mevalonate, the immediate product of HMG-CoA reductase, and GGPP, the precursor of geranylgeranylated proteins, but not FPP, the precursor of farnesylated proteins, or squalene, the late metabolite of the cholesterol synthesis pathway, prevented the effect of atorvastatin on DKK-1 expression by ECs (Fig. 2), suggesting a crucial role of protein geranylgeranylation on DKK-1 expression. Similarly, fluvastatin decreased DKK-1 in HUVECs by inhibiting geranylgeranylation, which suggests a class effect of statins on DKK-1 regulation (Supplementary Fig. 1B). To further verify this hypothesis, cells were treated with FTI-277 or GGTI-286, selective inhibitors of farnesyltransferase or geranylgeranyltransferase I, respectively. Our results showed that only the treatment with GGTI significantly inhibited DKK-1 (Fig. 2). Inhibition of geranylgeranylation by GGTI was confirmed by assessing ungeranylated RAP1A by immunoblotting (Supplementary Fig. 2). Because the Rho, Rac and cdc42 proteins of the Rho subfamily of GTPases have been involved as mediator of the vascular pleiotropic effects induced by statins^26^, we investigated the role of the Rho family members on DKK-1 expression by means of selective inhibitors. Treatment of cells with the Rho-associated protein kinase inhibitors Y-27632 and H-1152, the Rac1/2 inhibitor NSC23766, and the Cdc42 inhibitor ML-141, resulted in a significant reduction of DKK-1 release (Fig. 3). Taken together, these results suggest that mevalonate blockade suppresses DKK-1 via inhibition of the Cdc42, Rho and Rac geranylgeranylated proteins.

Further studies have demonstrated molecular links between the RhoGTPases and the NFκB pathway^27^ and a functional crosstalk between NF-kB signaling and Wnt/β-catenin signaling^28,29^. For example, Swarnkar *et al*. found that NF-kB activation inhibits osteogenic markers and stimulates the anti-osteogenic factor DKK-1^30^. Therefore, we tested the hypothesis that atorvastatin may affect DKK-1 expression by interfering with NF-kB activity. However, in our experimental conditions, chemical NF-kB inhibitors did not influence DKK-1 (Supplementary Fig. 3).

### DKK-1 mediates some statin effects in EC

**Figure 4:**
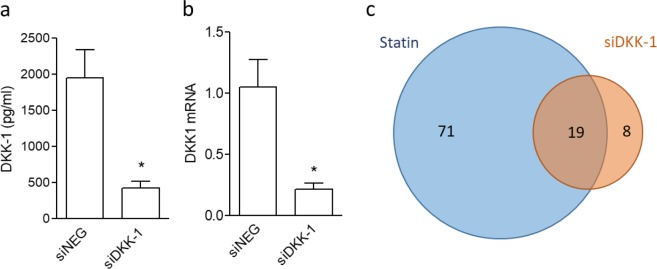
DKK-1 gene knockdown reduces DKK-1 in HUVECs. HUVECs were transfected with negative siRNA (siNEG) or with DKK-1 siRNA (siDKK-1) and then analysed for DKK-1 antigen release (**a**) and DKK-1 mRNA levels (**b**). Values are the mean ± SEM of 3 individual experiments. *p < 0.01 vs. control siRNA-treated cells. (**c**) Comparison of the effects of DKK-1 silencing and statin treatment on endothelial cell secretome using a Venn diagram generated with the differentially abundant proteins.

To identify the downstream effects of DKK-1 downregulation mediated by statin we adopted a combined strategy based on proteomics and DKK-1 gene silencing. First, we demonstrated that treatment of HUVECs with siRNA against DKK-1 (siDKK-1) resulted in a 76% reduction of DKK-1 mRNA and antigen secretion in comparison with cells treated with a negative siRNA (siNEG) (Fig. 4a and 4b). In our experimental conditions DKK-1 silencing does not affect cell viability and cell proliferation in HUVECs (Supplementary Fig. 4).

Then, we analysed the endothelial secretome derived from DKK1-silenced cells in respect to siNEG-treated cells, and compared the results with those obtained from the analysis of control and statin-treated cells in order to identify unique and common targets. To perform this analysis we employed a label free mass spectrometry based method (LC-MS^E^), that allows us to simultaneously achieve a qualitative and quantitative analysis of the protein content in the endothelial cells secretome^21^.

Proteins identified in the secretome in the different experimental conditions are reported in details in Supplementary Table S1 and S2. After treatment with siDKK-1, 6 proteins resulted to be significantly more secreted and 21 less secreted in respect to siNEG-treated cells (Table 1).

**Table 1:** List of differentially abundant proteins in the secretome of HUVECs treated with siDKK-1 identified by LC-MS^E^.

Accession	Description	Unique peptides/ Total peptide count	Confidence score	siDKK-1		Atorvastatin	
				fold siDKK-1/siNEG	pvalue	fold atorva/control	pvalue
**Proteins with an opposite behavior in respect to statin-treated cells**							
Q9UNN8	Endothelial protein C receptor	3/3	20,46	1,289	0,00058	0,638	6,79E-07
P49327	Fatty acid synthase	2/2	11,06	1,312	0,00014	0,471	2,05E-05
P26022	Pentraxin-related protein PTX3	2/2	13,05	2,136	1,3E-08	0,359	4,33E-06
P11142	Heat shock 71 kDa protein	14/26	221,77	1,207	9,5E-04	0,433	0,000152
P08238	Heat shock protein HSP 90-beta	12/24	188,89	1,209	0,00799	0,519	9,56E-06
**Proteins with the same behavior obtained with statin-treated cells**							
Q16270	Insulin-like growth factor-binding protein 7	8/8	57,56	0,796	0,00493	0,564	0,001956
Q8NBS9	Thioredoxin domain-containing protein 5	11/11	75,58	0,701	3,6E-05	0,599	0,000105
P43121	Cell surface glycoprotein MUC18	2/2	11,17	0,719	0,00024	0,585	3,33E-05
P30101	Protein disulfide-isomerase A3	18/19	140,69	0,814	0,00306	0,674	0,000733
P29966	Myristoylated alanine-rich C-kinase substrate	2/2	12,35	0,582	0,00302	0,437	0,060377
P29279	Connective tissue growth factor	8/16	114,37	0,776	0,00020	0,736	0,000162
P21810	Biglycan	10/12	92,66	0,607	0,00011	0,600	1,77E-05
P14625	Endoplasmin	10/11	67,74	0,812	0,01037	0,621	0,007624
P11021	78 kDa glucose-regulated protein	19/22	176,57	0,743	1,6E-06	0,637	0,00019
P09486	SPARC	10/10	91,66	0,827	3,8E-05	0,577	2,68E-06
P08253	72 kDa type IV collagenase MMP2	5/5	30,86	0,572	5,3E-06	0,799	0,000777
P07996	Thrombospondin-1	59/63	635,11	0,763	1,2E-04	0,682	8,2E-05
P06753	Tropomyosin alpha-3 chain	2/8	57,15	0,810	1,1E-05	0,575	2,84E-05
P05121	Plasminogen activator inhibitor 1	18/18	189,33	0,403	1,4E-06	0,816	0,00607
P04792	Heat shock protein beta-1	10/10	79,48	0,829	4,2E-03	0,644	0,000994
P02751	Fibronectin	56/56	478,89	0,820	0,00012	0,573	8,94E-05
P00491	Purine nucleoside phosphorylase	2/2	11,59	0,743	0,00020	0,571	3,18E-06
P13797	Plastin-3	10/10	61,67	0,822	1,2E-05	0,456	8,36E-05
P10909	Clusterin	5/5	33,41	1,304	0,00056	1,944	6,71E-05
**Proteins modulated by siDDK-1 only**							
P04275	von Willebrand factor	4/4	22,66	0,755	0,00028	1,001	0,960219
P98160	Basement membrane-specific heparan sulfate proteoglycan core protein	59/60	440,06	0,695	2,5E-05	1,081	0,097248
P61769	Beta-2-microglobulin	2/2	25,87	0,704	3,5E-03	0,997	0,984152

A comparison of the list of proteins modulated by siDKK-1 with those modulated by atorvastatin, led us to identify 19 proteins with a common behavior (Fig. 4c). In particular, only one protein, namely clusterin, was commonly enhanced by the two treatments, and 18 were reduced by both DKK-1 silencing and atorvastatin treatment. Five proteins were oppositely regulated by statin and DKK-1 silencing (i.e., pentraxin 3, PTX3), as reported in Table 1.

**Figure 5:**
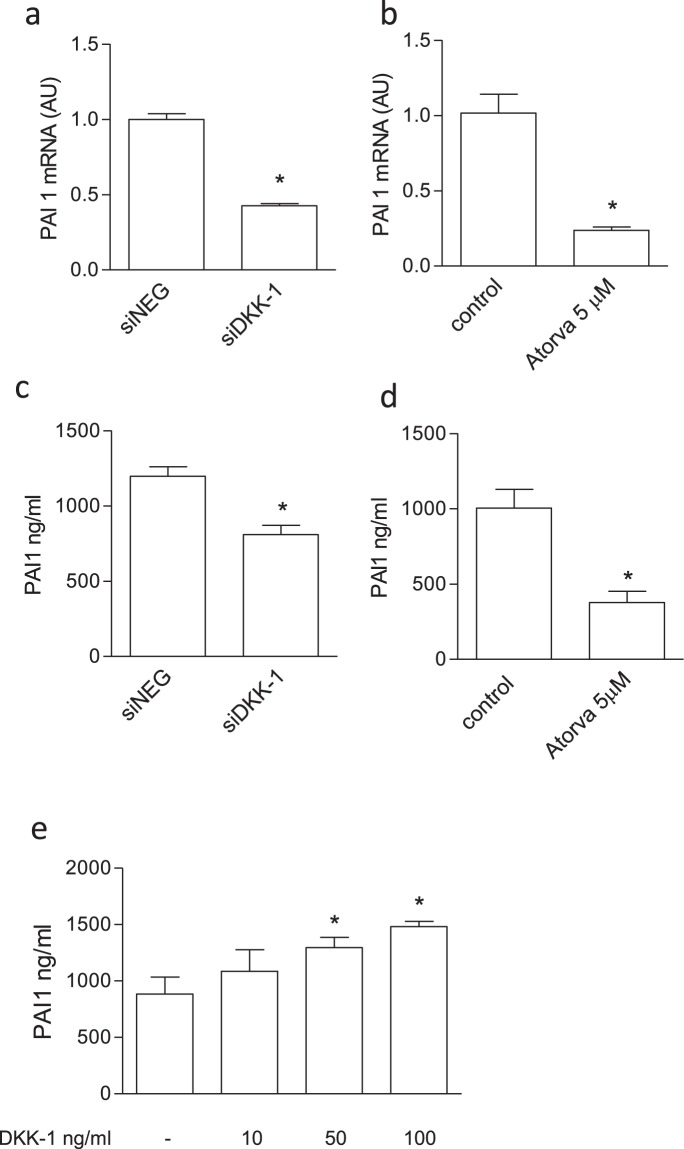
Validation of proteomics results regarding PAI-1 on endothelial cells treated with atorvastatin (Atorva) or by DKK-1 silencing as described in the Material and methods section (n = 3). Effect on PAI-1 mRNA levels exerted by siDKK-1 (**a**) or atorvastatin (**b**). PAI-1 was evaluated at antigen levels by ELISA in cells treated with siDKK-1 (**c)**, atorvastatin (**d**) or recombinant DKK-1 (**e**). The data are expressed as ng/ml of PAI-1. Values are the mean ± SEM of 3 individual experiments.*p < 0.05 *vs* siNEG or control cells.

**Figure 6:**
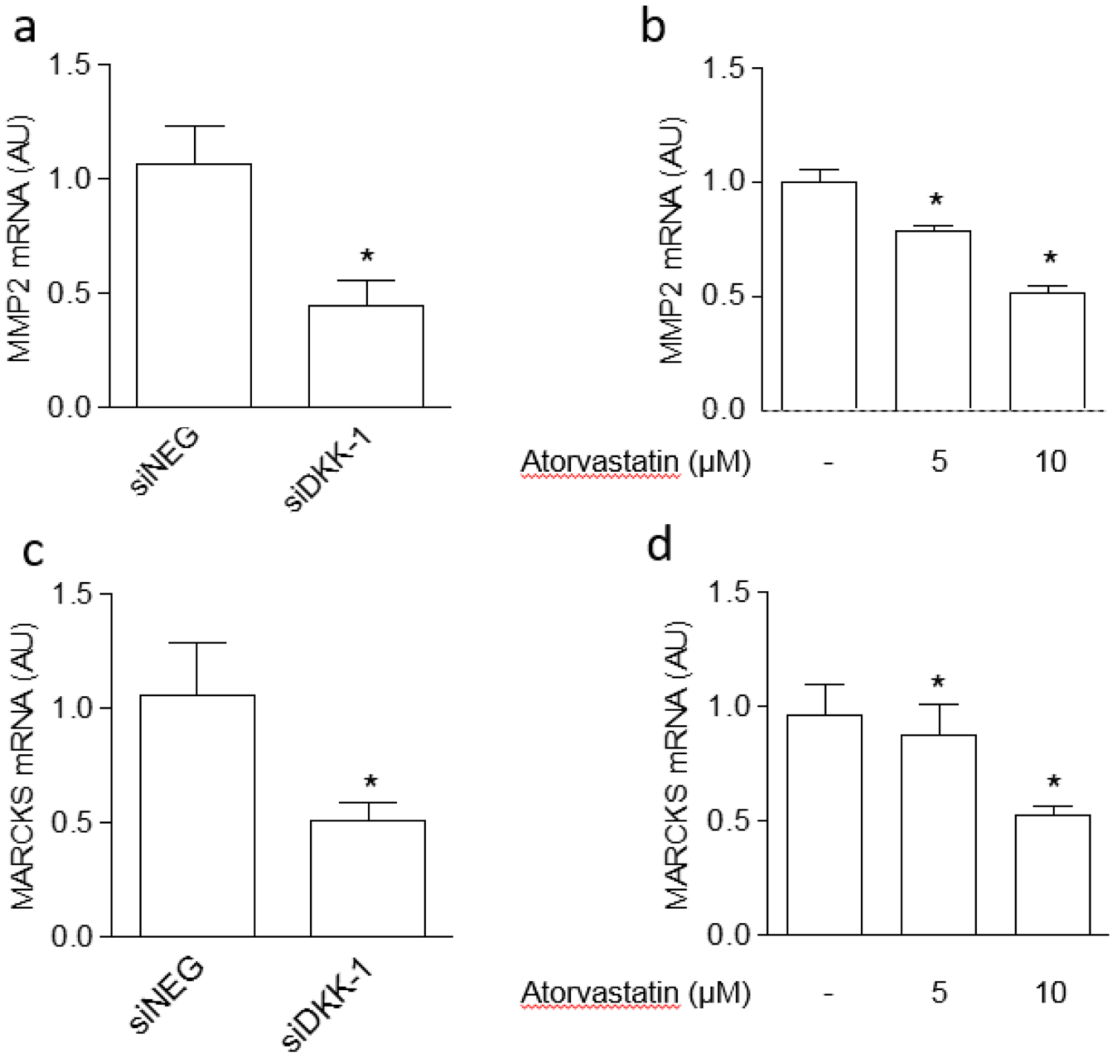
Validation of proteomics results regarding MMP-2 and MARCKS on endothelial cells treated with atorvastatin or by DKK-1 silencing. Effect on MMP-2 and MARCKS mRNA levels exerted by siDKK-1 (**a**,**c**) or atorvastatin (**b**,**d**). Values are the mean ± SEM of 3 individual experiments.*p < 0.05 vs siNEG or control cells.

**Figure 7:**
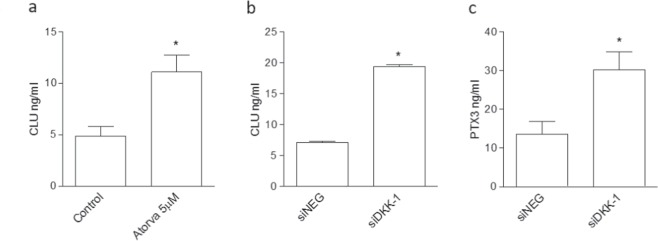
Validation of proteomics results regarding clusterin (CLU) and PTX3 on endothelial cells treated with atorvastatin or by DKK-1 silencing. (**a**) Effect of treatment with atorvastatin (**a**) and siDKK-1 on CLU antigen release. (**c**) Effect on PTX3 antigen exerted by siDKK-1. Values are the mean ± SEM of 3 individual experiments. *p < 0.05 vs siNEG-treated cells.

Some proteomics results were validated at mRNA level, by means of qRT-PCR, or at protein level, by means of specific ELISAs. Validation analysis confirmed, for example, that plasminogen activator inhibitor type 1 (PAI-1) was inhibited by both treatments, and it was increased after treatment with recombinant DKK-1 (Fig. 5). Further, both MMP-2 and MARCKS were modulated in the same manner by DKK-1 silencing and atorvastatin (Fig. 6). In addition, we found that clusterin release was increased by both statin treatment and DKK-1 silencing, while PTX3 secretion was enhanced by DKK-1 silencing (Fig. 7) and reduced by atorvastatin, as previously reported^31^.

**Figure 8:**
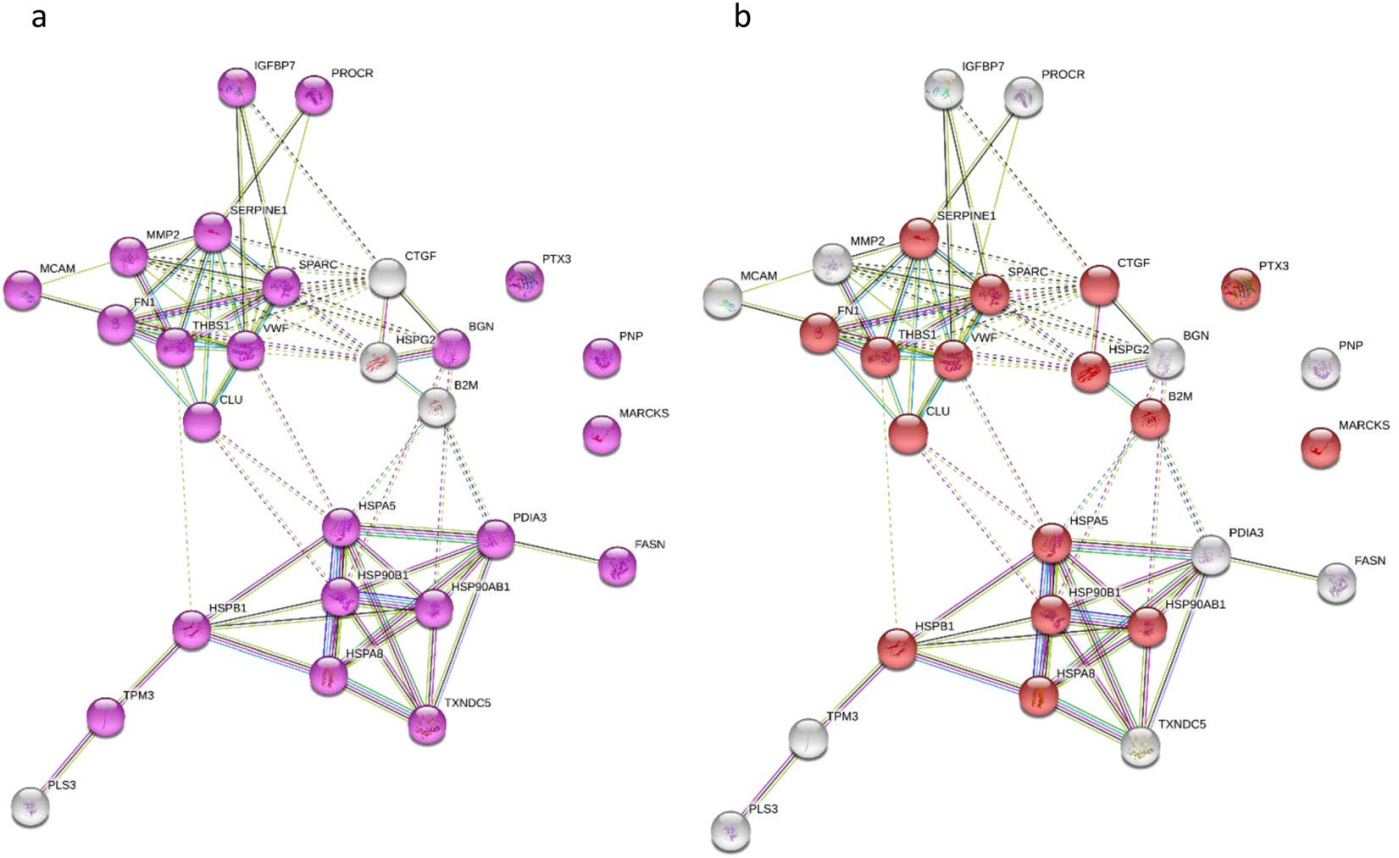
Gene ontology analysis of secreted proteins modulated by DKK-1 silencing in the cellular component and molecular function categories visualised with STRING. (**a**) Within the cellular component category the GO term related to Extracellular region resulted overrepresented (purple circles). (**b**) In the molecular function category the GO term protein binding was associated with the highest number of proteins (red circles). Detailed results are reported in Supplementary Table S3.

**Figure 9:**
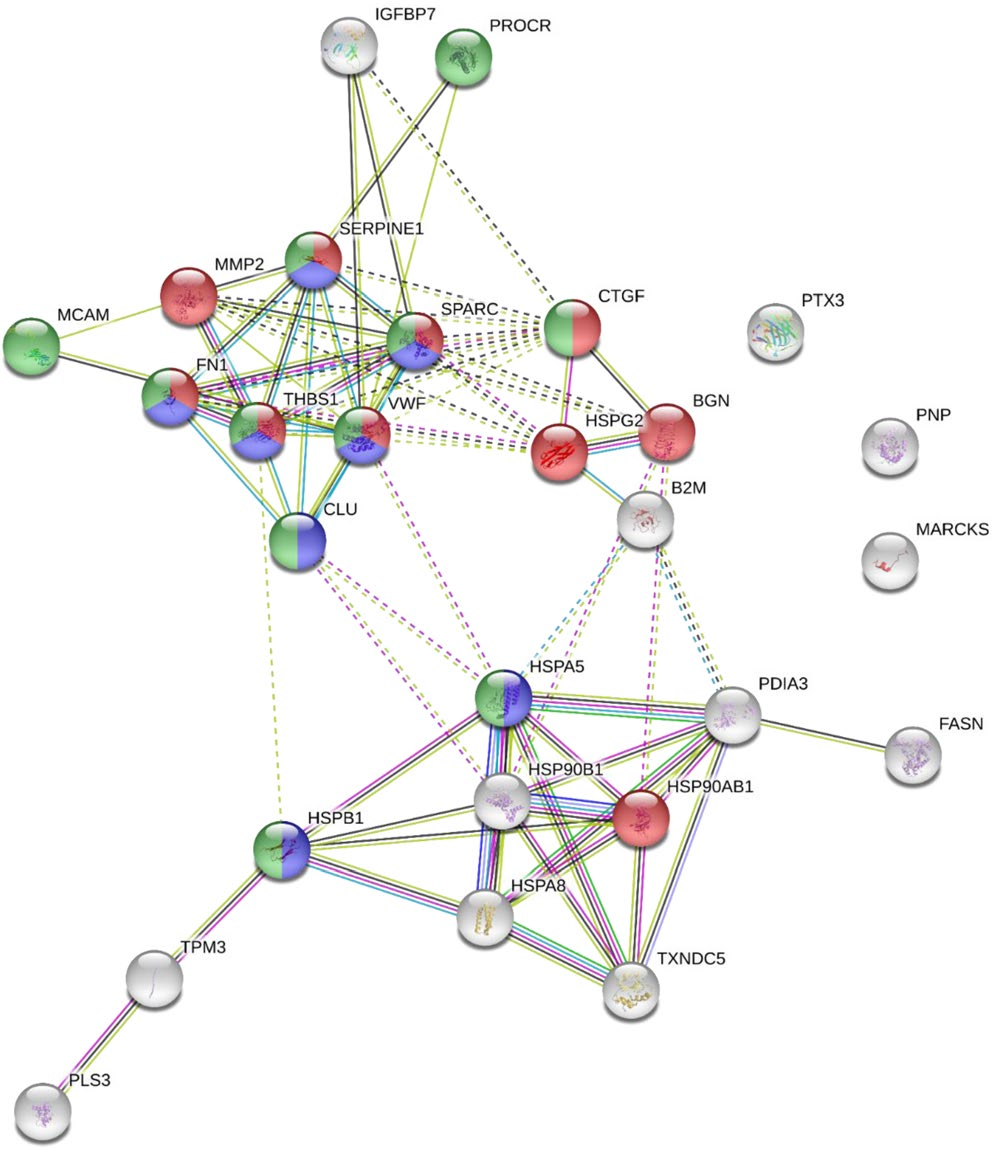
Biological processes of secreted proteins modulated by DKK-1 silencing visualised with STRING. Considering the biological processes category the enriched terms were response to wounding (green circles), extracellular matrix organization (red circles) and platelet activation (blue circles). Detailed results are reported in Supplementary Table S3.

Proteins modulated by siDKK-1 were analysed with STRING for evaluation of protein-protein interactions and gene ontology analysis in order to characterise their localization and function. As shown in Fig. 8, modulated proteins are mainly localised in the extracellular region (Fig. 8a, Supplementary Table S3), and the most over-represented term in the molecular function category is protein binding (Fig. 8b, Supplementary Table S3). Regarding the biological process category, the analysis of the most over-represented terms revealed that 10 proteins are involved in extracellular matrix organization, 11 in response to wounding and 8 in platelet activation (Fig. 9, Supplementary Table S3). Similar results were obtained performing the Gene Ontology analysis using a background containing only secreted proteins (Supplementary Table S4).

Of note, the proteins modulated by DKK-1 silencing are potentially responsible for much of the effects of statin on extracellular matrix organization (14 proteins, p = 1.19E-06 among statin-modulated proteins), on platelet activation response (29 proteins, p = 1.54E-13 among statin-modulated proteins), and on response to wounding (19 proteins, p = 1.54E-13 among statin-modulated proteins).

### Effect of atorvastatin on DKK-1 levels in plasma of cholesterol-fed rabbits

Since DKK-1 is a secreted protein whose levels are increased in experimental and clinical atherosclerosis^6^, to address whether statin-mediated DKK-1 regulation also occurs *in vivo*, we evaluated DKK-1 levels in plasma of cholesterol-fed rabbits treated with or without atorvastatin 1 mg/kg per day for 6 days. The semi-quantitative evaluation of DKK-1 performed by immunoblotting analysis on these samples showed a trend towards reduced levels of DKK1 in animals treated with atorvastatin compared to controls, although the difference did not reach statistical significance (Supplementary Fig. 5).

## Discussion

This study shows that DKK-1 represents a novel target of statins in primary human vascular cells (endothelial cells and aortic smooth muscle cells), and that it potentially mediates several of the pleiotropic effects exerted by this drug. Indeed, we found that atorvastatin downregulates DKK-1 protein and mRNA expression through the inhibition of Cdc42, Rho and Rac geranylgeranylated proteins, and that DKK-1 itself mediates, at least in part, statin effects on ECs. It directly regulates the expression of molecules involved in a plethora of biological functions, thus expanding its role, which has been so far restricted mainly to cancer^5,32^. Indeed, in two previous papers, Rachner *et al*.^23^ and Göbel *et al*.^22^ circumstantiated the effects of statins in combination with amino-bisphosphonates on DKK-1 expression in breast cancer cell lines. The rationale for these studies derives from the potential role of DKK-1 as promoter of osteolytic bone lesions through the inhibition of osteoblast functions and, therefore, as a potential adverse marker in multiple cancers. The authors showed that the suppression of DKK-1 by statin and amino-bisphosphonates inhibited the ability of breast cancer cells to block WNT3A-induced production of alkaline phosphates and bone-protective osteoprotegerin in preosteoblastic C2C12 cells.

In this study we took advantage of a proteomics-based approach to compare statin and DKK-1 effects in order to identify unique and common targets. Indeed, rapid recent progress in proteomics instrumentation and software have led to a marked decrease in the duration of a typical proteomics experiment, enabling analysis of thousands of proteins, and thus opening an unexplored opportunity to apply proteomics to many cellular proteomes for cross-comparison^33^. As described in our recent review, proteomics is also useful to understand the effects of a drug on its protein targets and shed light on the cellular mechanisms resulting in the observed phenotype^24^. Herein, proteomics allowed us to identify proteins uniquely or commonly regulated by statin and DKK-1, the latter representing the 21% of statin regulated proteins. Further, a functional interpretation of the identified proteins obtained by searching the Gene Ontology (GO) annotations for over-represented terms in the “biological process” category, revealed that DKK-1, besides being a downstream target of statin, is a potential mediator of the extracellular matrix organization, platelet activation and response to wounding processes induced by statin.

The effects exerted by DKK-1 on the endothelial cell proteome are not attributable to any perturbations of the cell viability or proliferation. Among the endothelial proteins commonly regulated by DKK-1 and atorvastatin we found clusterin, also known as apolipoprotein J (apoJ), which is considered one of the most important extracellular chaperones ever found^34^. It is involved in a broad range of physiological and pathophysiological functions, where it exerts mainly a cytoprotective role^34^, conferring, for example, resistance to endothelial cells against TNF-α-induced apoptosis^35^. Further, a potential anti-atherogenic role of exogenously administered clusterin/apoJ, partially attributable to an increased cholesterol efflux from foam cells, was demonstrated in apolipoprotein E-deficient mice and monkeys^36,37^. The only study available until now in endothelial cells, precisely in primary porcine brain capillary endothelial cells, suggests an importance of clusterin/apoJ in the production of amyloid precursor protein and Amyloid-β peptides transport in the brain^38^. Thus, our findings deserve further investigations in order to understand the role of clusterin/apoJ in human endothelial cells.

In the attempt to address the potential implications of the inhibitory effect observed *in vitro*, we performed a semi-quantitative evaluation of DKK-1 by immunoblotting analysis of plasma samples from rabbits treated with atorvastatin 1 mg/kg per day for 6 days, which were available from a previous study^39^. This dose of atorvastatin may be considered clinically relevant, since the administration of a dose 5 times greater, under comparable experimental conditions, resulted in atorvastatin-equivalent concentrations (mean total plasma concentrations of atorvastatin plus active metabolites) comparable to the concentrations detected in plasma of individuals treated with daily doses of 5–20 mg atorvastatin^39,40^. Herein, we found that the administration of atorvastatin was associated with a trend towards reduced levels of DKK1 in animals treated with atorvastatin compared to controls. Based on these observations, we believe that statin-mediated DKK-1 regulation can also occurs *in vivo*. This observation, however, needs further investigation and should be interpreted with caution because of its preliminary nature.

Interestingly, once released, a substantial part of circulating clusterin/apoJ associates to lipoproteins, mainly to HDL, where it suggested to play a relevant role in maintaining their qualitative properties^41^. Indeed, Riwanto *et al*. demonstrated that reduced HDL-associated clusterin in patients with coronary artery diseases (CAD) contributes to impair the anti-apoptotic capacity of HDL itself^42^. Conversely, the combined statin/niacin therapy increases levels of clusterin/apoJ in the HDL3 subpopulation of CAD patients, reverting the lipoprotein profile to the one more closely resembling that of HDL3 in healthy control subjects^43^.

Regarding the discovery of clusterin/apoJ regulation by DKK-1, it is worth highlighting that, if on the one hand it is well known that this protein is regulated by a wide variety of stimuli^44^, on the other hand, the signaling molecules responsible for the complex tissue-specific control of the clusterin/apoJ gene have been only partially identified and limited to the NF-κB or Src-Mek-Erk pathways^44,45^. Therefore, our results add a novel regulator to the list of signaling pathways which regulate clusterin/apoJ.

We also provide the evidence that DKK-1 is a mediator of the negative regulation exerted by atorvastatin on PAI-1. This secretory protein regulates, through potent inhibition of serine proteases tissue-type plasminogen activator (t-PA) and urokinase plasminogen activator, not only the fibrinolytic system, but also functions unrelated to this system, such as cellular processes, including cell adhesion, migration, and proliferation. It is well known that statin treatment of vascular cells results in decreased PAI-1 activity and synthesis, and increased tPA synthesis, expression, activity, and release^46^ (summarised in^47^). However, in the complex interplay of signaling pathways responsible for the effect of statin on PAI-1, which includes reduced expression of ROCK (Rho associated protein kinase) activity, a decrease of PTEN (phosphatase and tensin homolog), upregulation of Akt^48^, and lastly NF-κB, a role for DKK-1 has never been identified^49^.

In line with the evidence that statins inhibit the secretion of a broad spectrum of metalloproteases, in vascular cells and in foamy macrophages^26,50^, we confirm that MMP-2 was reduced in the secretome of endothelial cells, and we found that DKK-1 silencing mimicked the statin effect. Although the direct regulation of MMP-2 by DKK-1 in endothelial cells is of novelty, an effect of DKK-1 in the cell matrix remodeling has been observed in epithelial ovarian cancer cells cultured on three-dimensional collagen I gels, likely due to a transcriptional up-regulation of the membrane-tethered collagenase membrane type 1 matrix metalloproteinase^51^.

A novel candidate regulated by both statin and DKK-1 is represented by MARCKS (myristoylated alanine-rich C-kinase substrate), which, as indicated by the name, was originally identified as a major target of protein kinase C. A growing body of work now indicates MARCKS as a downstream effector in various processes including cytoskeletal control, regulation of the cell cycle, chemotaxis and motility, mediation of the inflammatory response, secretion and exocytosis, muscle spreading, neurological function, and development (reviewed in^52^). To this regard, MARCKS is usually considered a membrane-bound protein, which interacts with the plasma membrane by a combination of independent electrostatic interactions between the phospholipid bilayer and both the N-terminal myristoyl group and its effector domain. Being this interaction reversible, MARCKS can also shuttle back and forth between the membrane and the cytoplasm^53^. However, MARCKS has also been found in cell secretome of cancer cells^54–56^, therefore we cannot exclude a biological role for circulating MARCKS^56^. Recently, it has been indeed demonstrated, in a number of *in vivo* and *in vitro* studies, that a synthetic peptide identical to the N-terminus of MARCKS, affects MARCKS function related to vesicular transport, leukocyte degranulation and cell migration^57–59^. Lastly, an axolotl MARCKS-like protein has been identified as an extracellularly released factor that induces the initial cell cycle response during axolotl appendage regeneration^60^, raising the possibility that secretion of this protein may be linked to their ability to promote regeneration^56^.

Interestingly, we found an opposite effect of statin and DKK-1 silencing on PTX3, a major pattern recognition molecule belonging to the superfamily of pentraxins, which plays important roles as modulator of the immune-inflammatory responses^61,62^. While we confirmed our previous observation on the capacity of statins to inhibit the expression of PTX3 in vascular cells^31^, we found that DKK-1 silencing behaves in an opposite way upregulating PTX3. This suggests that DKK-1 is indeed a positive regulator of PTX3 and that statin overcomes it resulting in PTX3 inhibition through alternative ways. Of note, we add DKK-1 to the list of the signaling molecules involved in the regulation of PTX3, which actually only includes NF-kB and JNK in alveolar epithelial cells stimulated with TNFα^63^ and PI3K/Akt in endothelial cells treated with high-density lipoproteins^64^.

In conclusion, we show here the potential of proteomics to identify drug targets *in vitro*, a process called drug target deconvolution, that is, the action aimed at identifying the full spectrum of protein targets regulated by a bioactive molecule and the cellular phenotype that it induces. Of relevance, the identification of the proteins targeted by a drug could not only inform in advance about its therapeutic potential, but also predict its possible adverse effects through the identification of potential toxicity targets. Indeed, a large body of postgenomic biological research has progressively revealed that many effective drugs, such as statins, act on multiple rather than single protein targets, potentially having functional roles that go beyond their intended effects^26^. Finally, proteomics, through the analysis of protein networks, is able to reveal pathways affected by a drug, leading to the identification of molecules which mediate drug action.

## Materials and Methods

### Materials

The geranylgeranyl transferase I inhibitor GGTI-286, the farnesyl transferase inhibitor FTI-276, the cell permeable IkB kinase inhibitor peptide and its inactive control peptide, the NF-kB inhibitor SN50, the SN50M inactive control peptide, the Rac inhibitor NSC23766, the Rho-associated protein kinase (ROCK) inhibitors, Y- 27362 and H1152, and fluvastatin were purchased from Calbiochem (San Diego, CA, USA). Atorvastatin, mevalonate, squalene, geranylgeranyl pyrophosphate (GGPP), farnesyl pyrophosphate (FPP), Bay 11–7082 were purchased from Sigma-Aldrich (St. Louis, MO, USA). The Cdc42 inhibitor, ML141, was purchased by TOCRIS (Bristol, UK). Human recombinant DKK-1 was from R&D Systems (Minneapolis, MN, USA). Antibody against RAP1A was purchased from Santa Cruz Biotechnology Inc. (Dallas, Texas, USA).

### Cell cultures and experimental conditions

Human umbilical vein endothelial cells (HUVECs), human aortic smooth muscle cells (AoSMCs), EGM-2 Medium (Endothelial Cell Growth Medium-2 Bullet Kit) and SmGM-2 Medium (Smooth Muscle Growth Medium-2 Bullet Kit) were purchased from Lonza Walkersville Inc. (Walkersville, MD, USA). Cells were cultured according to Lonza’s Clonetics™ instructions. Experimental procedures have been described in detail in previous investigations^31,46^. Briefly, confluent cells were incubated for 24 h in complete medium containing atorvastatin or fluvastatin, respectively dissolved in ethanol and dimethyl sulfoxide. Then, the cells were incubated for 16 h in serum-free and phenol-free medium containing statins, with or without mevalonate and isoprenoid intermediates for antigen, mRNA and proteomics analysis. The control cells were treated with the appropriate vehicles. Experiments using chemical inhibitors of the various signaling pathways were performed under the same experimental conditions.

### Real-time quantitative RT-PCR

Total cellular RNA was extracted and reverse transcribed (1 µg) as previously described^65^. Amplification of 18S ribosomal RNA was used to correct for fluctuations in input RNA levels and the efficiency of the reactions. Real-time qRT-PCR was performed in triplicate with 2.5 μL of cDNA incubated in 22.5 μL IQ Supermix containing primers and SYBRGreen fluorescence dye (Bio-Rad Laboratories, Milan, Italy) using the iCycler Optical System (Bio-Rad Laboratories, Milan, Italy). The sequences of primers used as normalizer were: 18S forward: 5′-CGG CTA CCA CAT CCA AGG AA-3′; 18S reverse: 5′-CCT GTA TTG TTA TTT TTC GTC ACT ACC T-3′. The sequences of PTX-3 primers were: forward TGC GAT TCT GTT TTG TGC TC; reverse TGA AGA GCT TGT CCC ATT CC. Other specific primers were purchased from QIAGEN (Hilden, Germany): DKK-1, QT 00009093; PAI-1, QT 00062496; MMP2, QT 00088396; MARCKS, QT 00092694; CLU-1, QT 00054460.

### Antigen assays

The concentration of DKK-1, clusterin, PTX3 in the cell supernatant was measured using specific immunoassays from R&D System (Minneapolis, Minnesota, USA). PAI immunoassay was from BIOMEDICA Diagnostics (Stanford, CT, USA).

### Cell proliferation and viability assays

Cell Proliferation Kit I (MTT) and Lactate Dehydrogenase (LDH) Activity Assay Kit were from Sigma-Aldrich (St. Louis, MO, USA).

### RNA interference and cell transfection

Validated high-performance purity grade small interfering RNAs (siRNA) against human DKK-1 (SI00098546) were synthesised by Qiagen Inc. using the HiPerformance siRNA design algorithm and a proprietary homology analysis tool; siRNA with a non-silencing oligonucleotide sequence that does not recognise any known homology to mammalian genes was also generated as a negative control^31^.

### Secretome analysis

For the analysis of cell secretome by label-free mass spectrometry, the conditioned media were collected from vehicle, statin-treated or silenced cells and processed as described^21^. Before tryptic digestion, the samples were dissolved in 25 mmol/L NH_4_HCO_3_ containing 0.1% w/v RapiGest SF, reduced with 5 mmol/L dithiothreitol, dissolved in 100 mmol/L NH_4_HCO_3_, at 60 °C for 15 min, and then carbamidomethylated with 10 mmol/L iodacetamide for 30 min at room temperature. Digestion performed overnight at 37 °C using sequencing grade trypsin (Promega, Milan, Italy) was stopped by the addition of 2% v/v TFA. Quantitative label-free LC-MS^E^ was performed as previously described^66,67^. The proteins were identified by searching a human species-specific UniProt database (release 2017.1; 20,201 entries), and quantified using Progenesis QIP for proteomics (v3.0, NonLinear dynamics, Newcastle upon Tyne, UK).

### Data mining

Data mining was performed the Search Tool for the Retrieval of Interacting Genes/Proteins (STRING 10.5) database, to create network of interactions among differentially abundant proteins^68^. In order to find out the enriched GO terms in the biological process, molecular function or cellular component categories that can be associated to the proteins in the network, we employed the enrichment widget of STRING, which calculates an enrichment P-value based on the Hypergeometric test and the method of Benjamini and Hochberg for correction of multiple testing (P value cut-off of <0.05).

### Immunoblotting analysis of DKK-1 in plasma of cholesterol-fed rabbits

The levels of DKK-1 were analysed by immunoblotting in plasma samples from cholesterol-fed rabbits treated with or without atorvastatin (1 mg/kg/day given by oral gavage for 6 days), which were available from a previous *in vivo* dose-finding experiment with atorvastatin^39^. Plasma samples were diluted in Laemmli buffer containing protease inhibitor cocktail (Sigma-Aldrich, St. Louis, MO, USA). Equal volume of plasma (40 µl of diluted plasma 1:100) were separated on 12% SDS-polyacrylamide gels, transferred to nitrocellulose membrane and processed as previously described^69^. The antibody used was a HRP-conjugated rabbit polyclonal antibody raised against the AA 207–247 portion of human DKK1 (ABIN678167 antibodies-online Inc., Atlanta, USA), which display a 95% homology with its rabbit counterpart, as verified by the alignment of protein sequence from UniProt, accession numbers O94907 and Q6PVU5. Bands were visualized by enhanced chemiluminescence using the ECL kit (Amersham Corp.) and acquired by a densitometer (GS800 Biorad). In each analysis a plasma sample was loaded as control for normalization. Bands detected by ECL were quantitated by densitometry of exposed film using image analysis software QuantityOne (version 4.5.2) from Biorad. Results obtained as ratio of density versus the sample used as normalization control are expressed as arbitrary unit.

### Statistical analysis

In *in vitro* experiments, data were expressed as mean values ± SEM and were statistically compared using Student’s t-test or ANOVA for repeated measures, followed by Tukey’s test (n = the number of individual experiments performed in duplicate), after normality assessment by Kolmogorov– Smirnov and Shapiro–Wilk tests. P values of <0.05 were considered significant. *In vivo* data were analyzed with Mann-Whitney non parametric test.

## Electronic supplementary material

Supplementary Information

Supplementary Table S1

Supplementary Table S2

Supplementary Table S3

Supplementary Table S4
