# Supplementary material for: Identification of DKK-1 as a novel mediator of statin effects in human endothelial cells
Source: Sci Rep. 2018 Nov 12;8:16671. doi: 10.1038/s41598-018-35119-7 (PMC6232108; doi:10.1038/s41598-018-35119-7)
Supplement: Supplementary file 1 — Supplementary Information [file 41598_2018_35119_MOESM1_ESM.pdf]

Identification of DKK-1 as a novel mediator of statin effects in human endothelial cells

Marta Pontremoli[1], Maura Brioschi[1], Roberta Baetta[1], Stefania Ghilardi[1], Cristina Banfi[1*]

[1]Centro Cardiologico Monzino, IRCCS, Milano, Italy

Supplementary information

LEGENDS TO SUPPLEMENTARY FIGURES

Supplementary figure 1. A) Atorvastatin reduces DKK-1 release in AoSMCs. Data are expressed as mean ±SEM of 3 individual experiments. * $p<0.001$ *vs* control cells. B) Fluvastatin reduces DKK-1 release in HUVECs, and this effect was prevented by GGPP, but not by FPP. * $p<0.01$ *vs* control cells; # $p<0.01$ *vs* statin treated cells. Data are expressed as mean ±SEM of 3 individual experiments.

Supplementary figure 2. Inhibition of geranylgeranylation by GGTI-286. Ungeranylated RAP1A was assessed by immunoblotting in HUVECs treated with the GGTI-286. For immunoblotting assay, cell proteins were dissolved in Laemmli buffer containing protease inhibitor cocktail (Sigma, Milan, Italy), and equal amounts (50 µg) were separated on 12% polyacrylamide gel. Following transfer to nitrocellulose, membranes were stained with MemCode reversible protein stain (Pierce Biotechnology, Cramlington, UK) according to the manufacturer's instructions to ensure equivalent loading of proteins. For each sample, data are reported as the ratio of band volume, after local background subtraction, versus the volume of the total proteins stained with MemCode, and they are expressed as arbitrary units (AU). Representative image of the immunoblotting (A), MemCode staining (B) and densitometric analysis (C) of 3 independent experiments. *$p<0.05$ vs control cells.

Supplementary figure 3. DDK1 release is not modulated by NF-kB. HUVECs were treated with increasing doses of BAY (A), the cell permeable IkB kinase inhibitor peptide IKK (B) and its inactive control peptide in-IKK (C), the NF-kB inhibitor SN50, D) and its inactive control peptide SN50M (E). Data are expressed as mean ±SEM of 3 individual experiments.

Supplementary figure 4. DKK-1 gene knockdown does not affect cell viability and proliferation in HUVECs. HUVECs were transfected with negative siRNA (siNEG) or with DKK-1 siRNA (siDKK-1)

and then analysed for LDH release in the medium (A) and MTT (B). Values are the mean±SEM of 3 individual experiments.

Supplementary figure 5. DKK-1 levels in plasma of control and atorvastatin-treated rabbits. New Zealand White cholesterol-fed rabbits were treated with or without atorvastatin 1 mg/kg per day for 6 days. Immunoblotting images of DKK1 in plasma samples from 7 control rabbits and 7 statin-treated rabbits are shown. Plasma sample from control rabbit (C1) has been loaded in all gels for normalization. Graph shows results of densitometric analysis of DKK-1 in control and atorvastatin-treated rabbits (n=7 per group) expressed as arbitrary units normalized for the plasma sample used as control. Data are median and interquartile range.

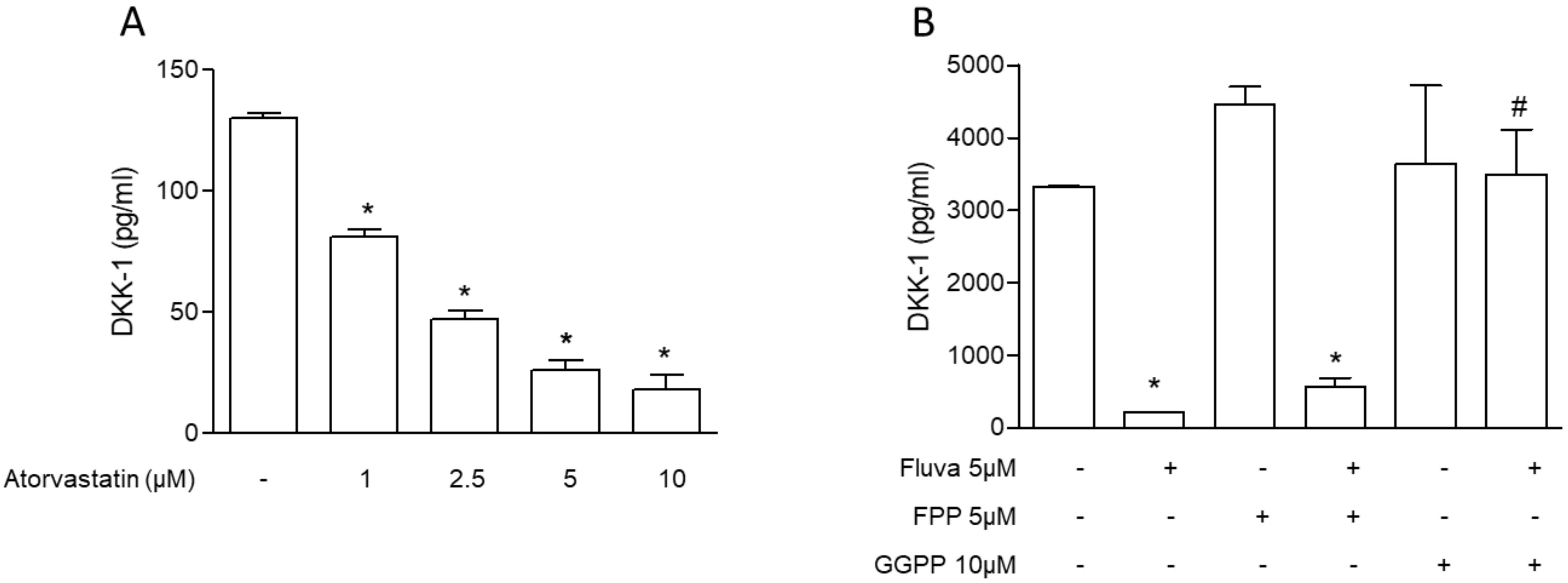

Supplementary Figure 1

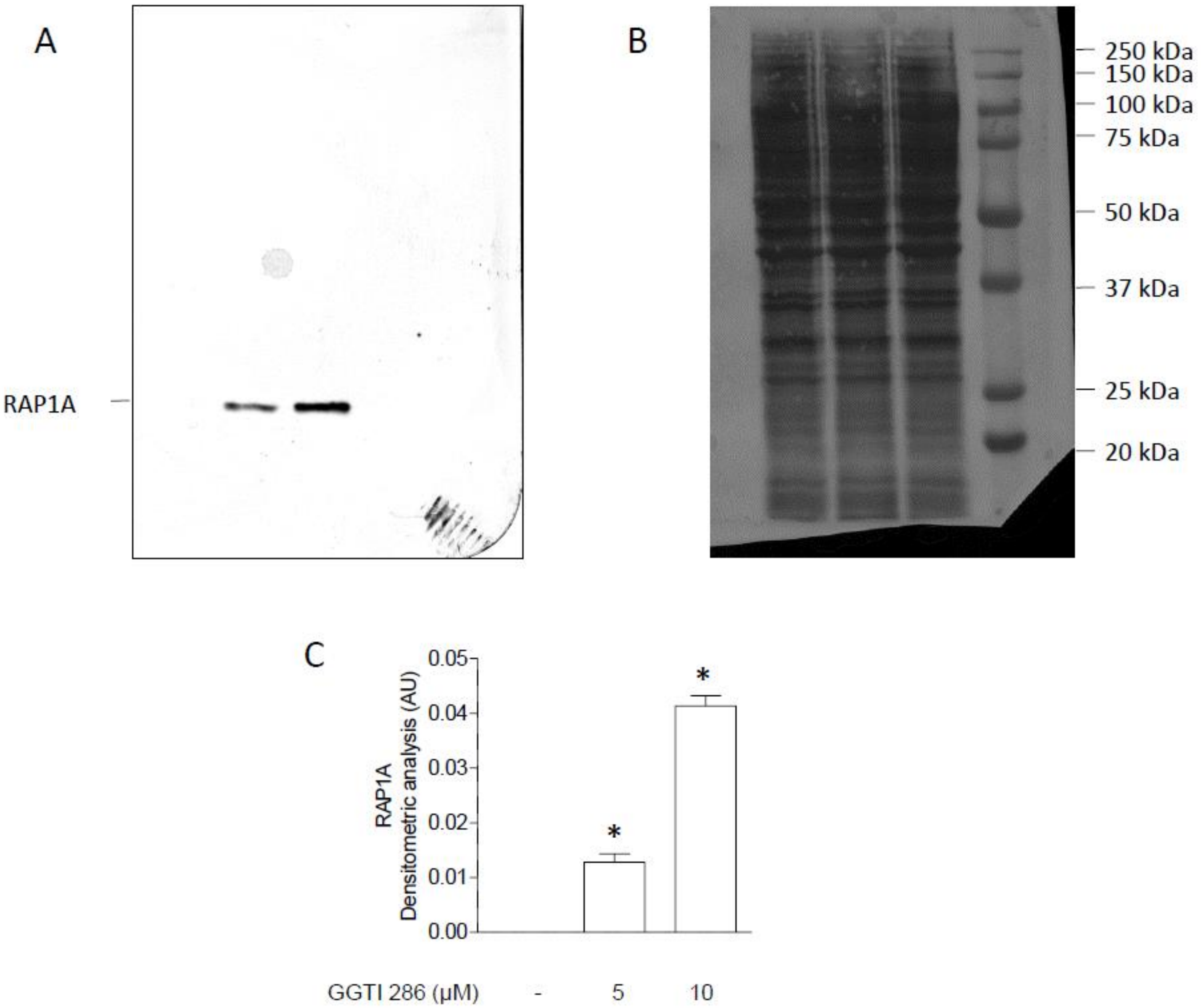

Supplementary figure 2

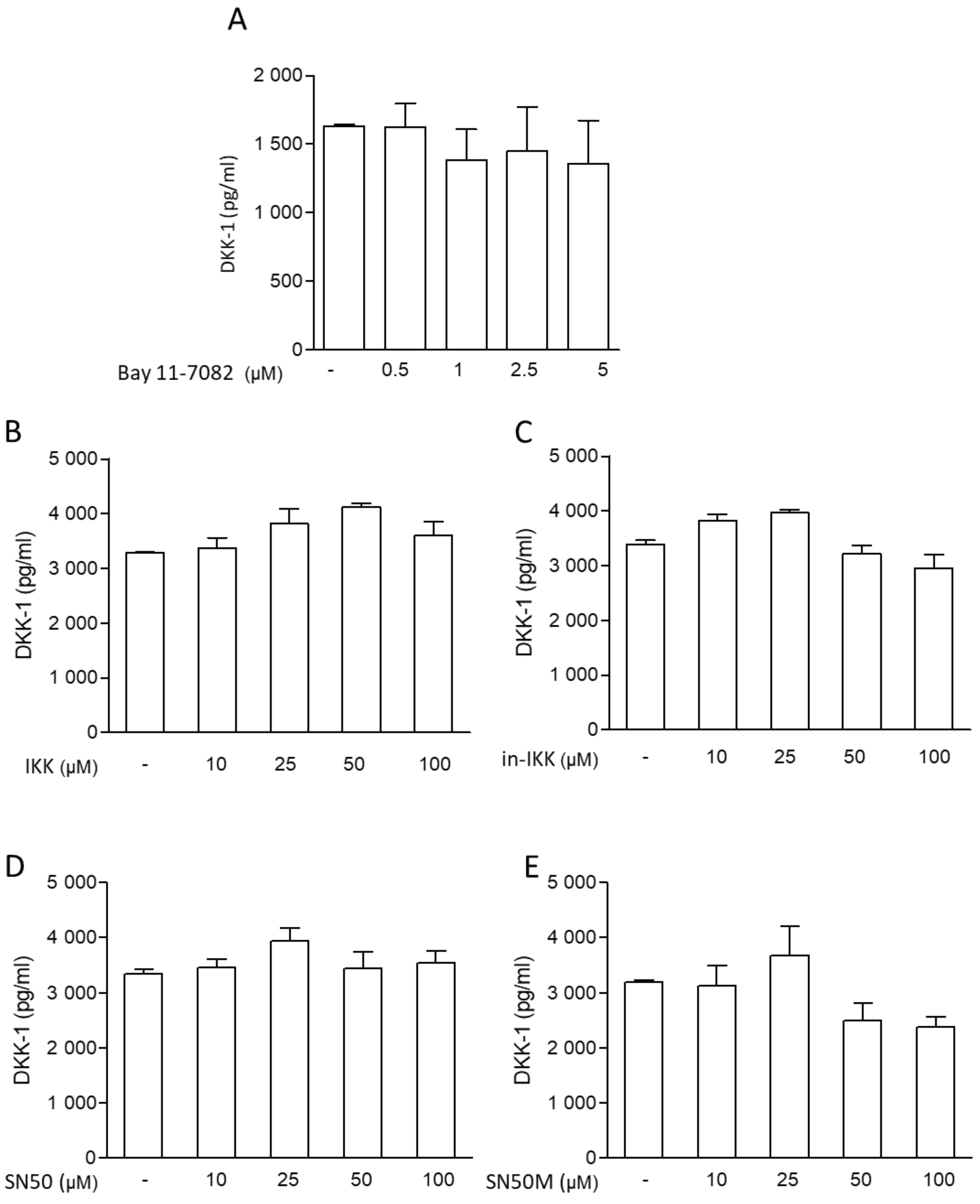

Supplementary Figure 3

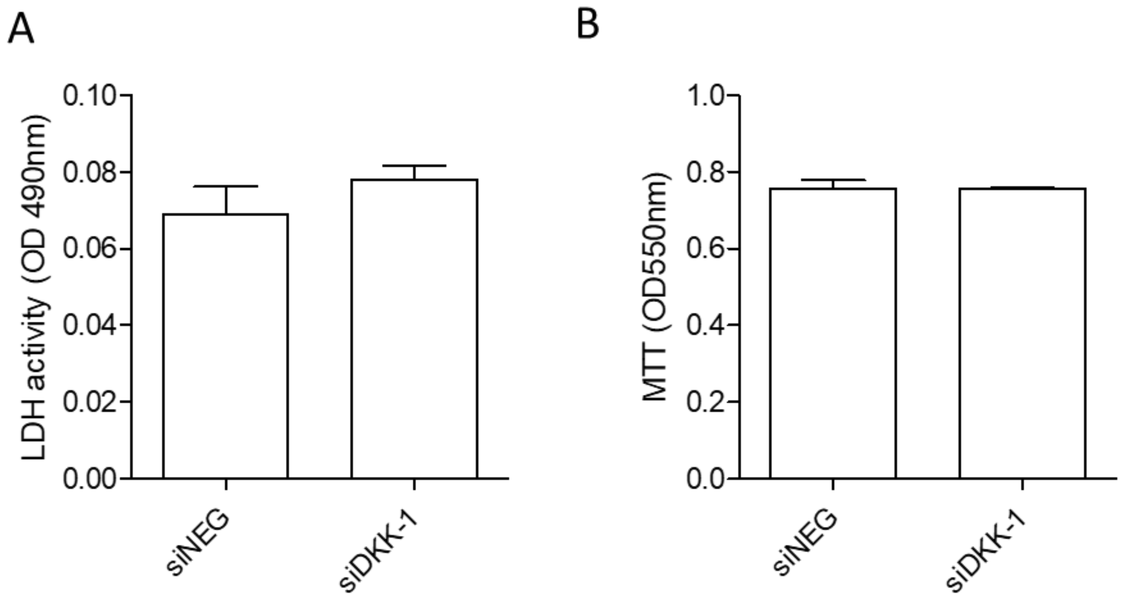

Supplementary Figure 4

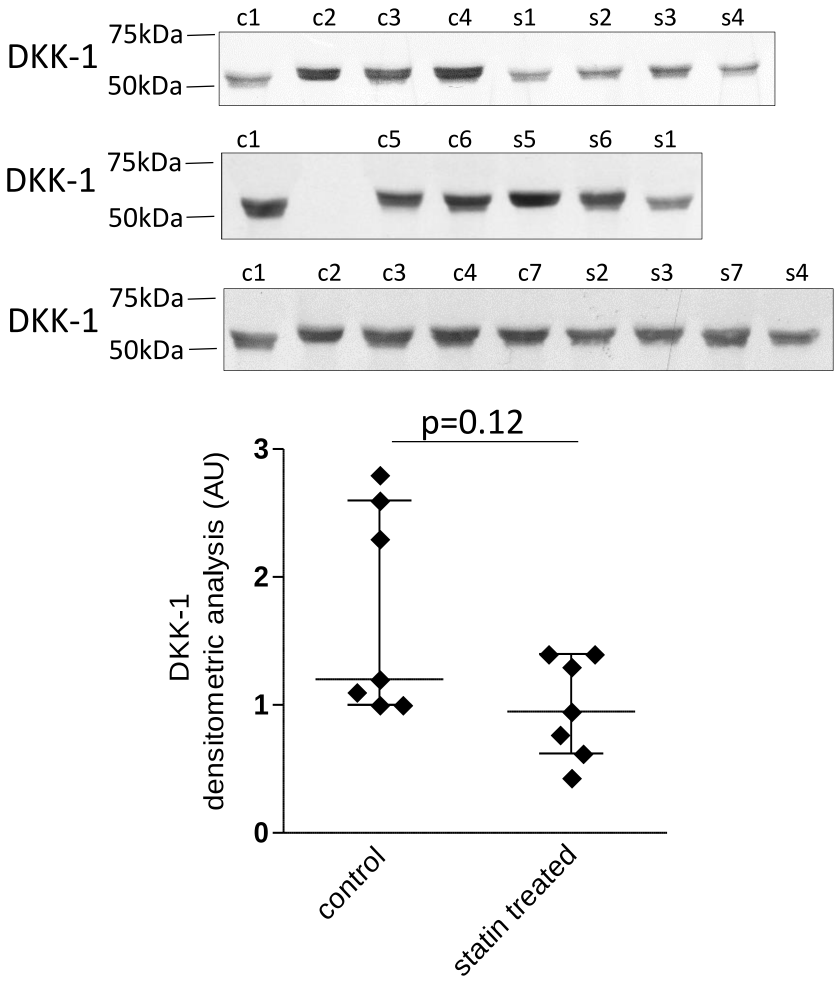

Supplementary Figure 5
